# The Evaluation of Antibacterial Coatings Against Commonly Found Pathogenic Bacteria in the Environment—Implications for Environmental Safety and Infection Prevention

**DOI:** 10.3390/pathogens14010013

**Published:** 2024-12-30

**Authors:** Wei-Chuan Chen, Mei-Hsiu Ho, Shin-Huan Yuan, Hui-Wen Chou, Chi-Chuan Chang, Chih-Hung Chen, Yusen Eason Lin

**Affiliations:** 1 Department of Medical Education and Research, Kaohsiung Veterans General Hospital, Kaohsiung 813414, Taiwan; wcchen1027@vghks.gov.tw (W.-C.C.);; 2 Department of Pharmacy and Master Program, Tajen University, Pingtung County 907101, Taiwan; 3 Shu-Zen Junior College of Medicine and Management, Kaohsiung 821004, Taiwan; 4 Center for Environmental Laboratory Services, National Kaohsiung Normal University, Kaohsiung 824004, Taiwan; 5 School of Medicine, National Cheng Kung University, Tainan 701401, Taiwan; em75010@email.ncku.edu.tw; 6 Department of Laboratory Medicine, Kaohsiung Medical University Hospital, Kaohsiung 807378, Taiwan; 7 Division of Hepato-Gastroenterology, Department of Internal Medicine, Kaohsiung Chang Gung Memorial Hospital, Kaohsiung 833401, Taiwan; totoro@cgmh.org.tw; 8 College of Medicine, Chang Gung University, Taoyuan 333323, Taiwan; 9 Center for Faculty Development, Kaohsiung Chang Gung Memorial Hospital, Kaohsiung 833401, Taiwan; 10 Graduate Institute of Human Resource and Knowledge Management, National Kaohsiung Normal University, Kaohsiung 802561, Taiwan

**Keywords:** antibacterial coating, infection prevention, environmentally friendly

## Abstract

Microorganisms, including pathogens that cause skin, respiratory, and urinary tract infections, are widespread in our environment. Despite routine cleaning with bleach and disinfectants, the transmission of pathogens still occurs, leading to potential infectious diseases. This study aimed to determine the antibacterial effect of two coating formulas against common environmental pathogens like *Escherichia coli*, *Staphylococcus aureus*, *Acinetobacter baumannii*, *Pseudomonas aeruginosa*, *Salmonella* sp., vancomycin-resistant *Enterococcus faecium*, and *Klebsiella pneumoniae*. The antibacterial coatings exhibited efficacy against these pathogens, with a bacterial reduction rate (R) greater than two, even after prolonged bleach cleaning. However, UV exposure may affect the efficacy of such coatings. The antibacterial coatings can eliminate the need for repetitive consumption of toxic detergents and reduce cleaning manpower, making them an environmentally friendly product for safety control and infection prevention.

## 1. Introduction

Healthcare-associated infections (HAIs) remain a critical global challenge, impacting millions of people annually and presenting a major risk to patient safety [1]. Pathogenic microorganisms, including *Staphylococcus aureus*, *Escherichia coli*, and *Acinetobacter baumannii*, are frequently encountered in healthcare environments. If not properly managed, these bacteria can survive on surfaces for extended periods, leading to skin, respiratory, and urinary tract infections [2]. These pathogens can be transmitted through contact with contaminated surfaces, ingestion, or inhalation, increasing the likelihood of infections, especially in healthcare settings. For instance, patients admitted to rooms previously occupied by individuals with methicillin-resistant *Staphylococcus aureus* (MRSA) or vancomycin-resistant *Enterococcus* (VRE) face significantly increased odds of acquiring these infections [3].

However, to combat the spread of harmful microorganisms, conventional disinfection methods often rely on chemical agents like chlorine, which can be corrosive to surfaces and produce harmful byproducts, such as trihalomethanes (THMs) and haloacetic acids (HAAs), which are linked to cancer and reproductive health risks [4]. Given these limitations, there is a growing demand for more sustainable and non-toxic antimicrobial solutions.

In response to the limitations of traditional disinfection methods, antimicrobial coatings have emerged as a promising solution for reducing bacterial colonization on surfaces, particularly in healthcare environments [2,5,6,7,8]. Among these, triclosan-based coatings stand out as an effective alternative. Triclosan is a broad-spectrum antibacterial agent that inhibits the enzyme enoyl-acyl carrier protein reductase (ENR), a key player in bacterial fatty acid synthesis, leading to the disruption of cell membrane formation and, ultimately, bacterial cell death [9,10]. By binding to ENR’s active site, triclosan prevents proper enzymatic function, halting fatty acid biosynthesis and killing the bacteria [11]. These coatings can be applied to various surfaces, such as metals, plastics, and textiles, offering long-term protection against bacterial growth and colonization [6]. This technology has already been successfully implemented in industries like household appliances, with companies like LG and Whirlpool integrating antimicrobial coatings into refrigerators and washing machines to improve hygiene.

Despite the established use of antimicrobial coatings in various sectors, their application in healthcare remains underexplored, particularly regarding their effectiveness on materials commonly found in medical settings, such as stainless steel. Stainless steel surfaces, prevalent in hospitals and clinics, are highly susceptible to bacterial contamination, making them ideal candidates for antimicrobial treatment.

This study evaluates the antimicrobial efficacy of two novel coatings, 23EV and 28SC, developed by a Kaohsiung-based steel plate manufacturer. These coatings, containing triclosan-based additives, were applied to stainless steel plates using an electrostatic coating method and were engineered to provide durable antimicrobial protection. In this study, we assessed the effectiveness of these coatings against common pathogens, including *Staphylococcus aureus, Pseudomonas aeruginosa*, and *Escherichia coli*, using protocols from the Japanese Industrial Standards Committee (JIS Z 2801:2010(E)) [12]. Additionally, we examined whether prolonged UV exposure and cleaning with bleach affected the coatings’ long-term efficacy.

## 2. Material and Methods

We followed the JIS Z 2801:2010(E) standard on determining the efficacy of bacterial reduction by washing off the bacterial inoculum from the surface with antibacterial coatings [12]. We also used swabs to remove any remaining bacteria on the surface of the coatings to determine whether residual bacteria can be recovered after 24 h of contact to determine the total reduction by antibacterial coatings.

### 2.1. The Anti-Bacterial Material Preparation

This study assessed the antimicrobial properties of two novel coatings, 23EV and 28SC, developed by a steel plate manufacturer based in Kaohsiung. The stainless steel plates were cut into 60 mm by 60 mm squares, serving as the test specimens. The two triclosan-based coatings were engineered onto the steel plates. The 23EV and 28SC formulations were designed to meet the environmental requirements of the European Union’s RoHS and REACH directives. Their antimicrobial efficacy has been verified by third-party institutions such as SGS, the Food Industry Research and Development Institute, and the Singapore TÜV SÜD PSB standard.

The coatings were applied to the stainless steel plates using an electrostatic coating method. First, the steel surfaces were thoroughly cleaned to remove contaminants, ensuring proper adhesion. A primer layer was applied to the plates’ top and back sides to improve bonding and corrosion resistance. The antimicrobial coatings were then evenly applied to the plates using a controlled electrostatic spraying technique. The coating thickness was standardized for consistency. After coating, the plates were cured in an oven to harden the antimicrobial layer, ensuring its durability and adherence to the surface. Finally, the plates were allowed to cool to room temperature.

The antimicrobial properties of these coatings are attributed to the integration of specialized triclosan-based additives within an eco-friendly surface passivation layer. These additives are uniformly distributed to form a robust antimicrobial film that can withstand cleaning or wiping under normal conditions.

Before each experiment, the coated and uncoated (control) steel plates were sterilized by lightly wiping them with ethanol-soaked gauze two to three times, followed by air drying. To ensure statistical reliability, triplicate samples of the coated and uncoated plates were used in each experiment.

### 2.2. Test Pathogens

The pathogens tested in this study were *Staphylococcus aureus*, *Escherichia coli*, *Acinetobacter baumannii*, multi-drug-resistant *Acinetobacter baumannii* (MDRAB), multi-drug-resistant *Staphylococcus aureus* (MRSA), *Pseudomonas aeruginosa*, *Salmonella* sp., vancomycin-resistant *Enterococcus faecium* (VREF), *Klebsiella pneumoniae*, and *Escherichia coli* with an ESBL feature.

### 2.3. Buffer Solution and Culture Medium Preparation

We followed the JIS Z 2801:2010(E) protocol to prepare the nutrient broth, SCDLP broth, and phosphate-buffered physiological saline. The blood agar plate (BAP) was used for bacterial enumeration.

### 2.4. Preparation of the Bacteria Solution

A platinum loop was used to remove the subculture from the medium, and then the subculture was suspended in 35 mL of sterile deionized water. The cells were washed twice by centrifugation at 4000 rpm for 10 min. The suspension was standardized by comparison with a McFarland No.1 standard (approximate density of 3 × 10^8^ CFU/mL). Then, this solution was diluted with 1/500 nutrient broth appropriately to reach the final concentration of approximately 2.5 to 10 × 10^5^ CFU/mL.

### 2.5. Inoculation and Incubation of the Test Plates

Each test plate was placed in a petri dish. A total of 0.4 mL of the bacteria solution was placed onto each test plate. The bacteria solution was then covered with parafilm so that the bacteria solution would be spread over the steel plate. Finally, the lid was placed on the petri dish. The petri dishes that contained the test plates were incubated (three control and three antibacterial test plates) at a temperature of 35 ± 1 °C and a relative humidity of ≥90% for 24 h ± 1 h.

### 2.6. Washing off the Inoculated Bacteria from the Coating Surface

The three Petri dishes with the test steel plates, which had been placed inside for over 24 h, were opened, and the parafilm was removed. First, the parafilm was put into a 120 mL sterilized measuring cup. Then, 10 mL SCDLP was used to rinse the antibacterial steel plates in the measuring cup. The same process was performed for the controls.

### 2.7. Swabbing the Bacteria from the Coating Surface

The swab method was used to collect the bacteria that remained on the surface of the test and control plates. A swab was used to firmly wipe the surface, covered by parafilm during incubation. The swab was then placed in 10 mL SCDLP. A total of 0.1 mL of the solution was taken for bacterial enumeration.

### 2.8. Bacterial Enumeration

A total of 0.1 mL of the washing fluid was placed in a test tube that contained 9.9 mL of phosphate-buffered physiological saline. Then, 0.1 mL of the mixture was dispensed and diluted into two flasks of BAP media. The media were incubated at 35 ± 1 °C for 48 h. After the incubation, the numbers of colonies in these BAP cultures were recorded

### 2.9. The Effect of UV Exposure on the Antibacterial Coating and Bacterial Reduction Rate

The antibacterial steel plates were first exposed to UV light for 500 h and then were used for bacterial reduction tests against *Salmonella*, MDRAB, and MRSA. The same procedures were followed as previously described.

### 2.10. The Effect of Sodium Hypochlorite on the Antibacterial Coating and Bacterial Reduction Rate

The antibacterial steel plates were rubbed with 0.06% sodium hypochlorite four times daily for two weeks. The plates were used for bacterial reduction tests against *Salmonella,* MDRAB, and MRSA. The same procedures were followed as previously described.

### 2.11. Calculation of the Bacterial Reduction Rate

This study used two bacterial reduction rates, R(JIS) and R(Total), for evaluation in this study. Based on the JIS 2801:2010 standard, the effectiveness of the anti-bacteria coating is determined by the bacterial reduction rate, R(JIS),

R(JIS) = 〔log(A/B)〕

where R is the value of the bacterial reduction rate, A is the average of the number of viable bacteria which were sluiced from the control plates after 24 h, and B is the average of the number of viable bacteria which were sluiced from the antibacterial test plates after 24 h.

According to the JIS Z 2801:2010(E) standard, the bacterial reduction rate must be greater than 2 to be recognized as a product with an antibacterial effect.

We also define an R(Total), total bacterial reduction, which includes the bacteria washed off from the antibacterial coating and the bacteria remaining on the coating surface.

R(Total) = 〔log(A + C)/(B + D)〕

where R is the value of the bacterial reduction rate, A is the average number of viable bacteria sluiced from the control plates after 24 h, B is the average number of viable bacteria sluiced from the antibacterial test plates after 24 h, C is the average number of viable bacteria swapped from the control plates after 24 h, and D is the average number of viable bacteria swapped from the antibacterial test plates after 24 h.

## 3. Results

### 3.1. Efficacy of the Antibacterial Coating

This study tested ten different bacteria to evaluate the effectiveness of the two antibacterial coatings—23EV and 28SC. The bacterial reduction rates, R(JIS) and R(Total), of both coatings were found to be greater than two, indicating a 99% reduction for all pathogens tested (Table 1a,b). Our data suggested that both coatings were effective in reducing bacterial colonization/attachment on the coating surfaces, and that both coatings were most effective against *K. pneumoniae*, with R values all greater than five (99.999% reduction). In general, 23EV was more effective against this pathogen than 28SC. On the other hand, 23EV was found to be less effective against *S. aureus*, while 28SC was less effective against MRSA and VREF (Table 1a,b).

Overall, the study suggested that both antibacterial coatings can effectively reduce bacterial contamination on surfaces, but their effectiveness may vary depending on the bacterium. These findings may be useful in developing targeted strategies for controlling the spread of specific bacterial pathogens in different environments, such as hospitals or other public spaces.

### 3.2. The Effect of Prolonged UV Exposure on the Efficacy of the Antibacterial Coatings

UV light is a commonly used surface disinfection method in healthcare facilities and public areas. This study determined the antibacterial efficacy under prolonged UV exposure (>500 h). Our findings showed that UV light compromises the antibacterial efficacy with decreased R values (Table 2). For 23EV, the R value decreased from 2.95 to 0.072 for MDRAB. For 28SC, the R value decreased from 2.82 and 2.12 to 1.135 and −0.836 for MDRAB and MRSA, respectively. Our results indicated that the efficacy of the antibacterial coatings was compromised by prolonged exposure to UV light.

### 3.3. The Effect of Bleach on the Efficacy of the Antibacterial Coatings

Bleach is also a commonly used disinfection method in healthcare facilities. This study determined the antibacterial efficacy under the effect of bleach (0.06% sodium hypochlorite). Results showed that the bacterial reduction rates were greater than two (Table 3). The data indicated that 23EV and 28SC had antibacterial efficacy against pathogens tested after exposure to 0.06% sodium hypochlorite.

## 4. Discussion

Many pathogenic bacteria in our environment may cause infections in humans. In addition, the overuse of antibiotics has accelerated the development of antibiotic resistance among bacteria. Conventional disinfection methods, such as chlorine-based solutions, use toxic materials for bacterial eradication. Thus, the need for non-toxic disinfection technology has emerged. The antibacterial coating may be a good candidate that can reduce the bacteria viability and morbidity yet be environmentally friendly [4,5,6,7,8,13].

The two coatings tested in this study exhibited antibacterial efficacy against ten pathogens. The antibacterial efficacy of the 23EV coating was better than 28SC. After the steel plates with antibacterial coatings were exposed to UV light for 500 h, the antibacterial efficacy of the coatings became unstable. This suggested that the efficacy of antibacterial coatings is compromised by prolonged UV light. The efficacy of such antibacterial coatings on outdoor applications may be affected, so we propose the use of such antibacterial coatings on indoor applications, such as rooms, walls, and facilities, to minimize the impact of prolonged UV exposures. However, the antibacterial efficacy was not affected by contact with 0.06% sodium hypochlorite solution. This may indicate that the antibacterial coatings can be used in hospital settings where chlorine disinfection is used as standardized procedure. Such cleaning practices may not compromise the antibacterial efficacy of the coatings.

Triclosan, the major component of the antibacterial coatings used in this study, has been shown to disrupt the proton-motive force (PMF) of bacterial cells by inhibiting the activity of the membrane-bound ATP synthase complex, which is responsible for generating ATP from the electrochemical gradient created by the PMF [11]. The disruption of the PMF by triclosan can lead to the depletion of cellular energy reserves and, ultimately, cell death, and has been observed in both Gram-positive and Gram-negative bacteria [10]. However, triclosan may pose potential toxicity to humans and the environment. For example, triclosan has been shown to disrupt hormone function, particularly in animal studies. Triclosan can interfere with the thyroid hormone, estrogen, and androgen systems, leading to adverse developmental, reproductive, and behavioral effects [14,15,16,17]. In the environment, triclosan can accumulate in waterways and soil, persisting for long periods and potentially harming wildlife. Triclosan is toxic to aquatic organisms, including algae, fish, and other aquatic invertebrates. In addition, when exposed to sunlight, triclosan can break down into harmful byproducts, including dioxins and other toxic compounds [7,18,19,20,21]. Due to these potential risks, triclosan has been banned or restricted in some countries, including the European Union and Canada. USFDA has issued a rule banning triclosan and other antibacterial agents in over-the-counter consumer antiseptic wash products [19]. However, the toxicity mentioned above for triclosan is mostly in free soluble forms. In our application, triclosan was mixed with oil paints as additives in the antibacterial coatings of surfaces. The release of free triclosan into the environment was minimal.

Our findings suggest that using materials with antimicrobial coatings effectively eliminates pathogenic bacteria and multiple drug-resistant pathogens, including MDRAB, MRSA, and VREF [22]. These coatings can be used proactively before the accessories are manufactured or as a reactive approach after the interior is furnished [5,22]. The use of antimicrobial coatings can reduce bacterial colonization and help prevent infections, especially in healthcare settings [6]. In addition, this prevention strategy can help reduce the spread of drug-resistant pathogens, which can be especially beneficial in hospitals and other healthcare facilities with high patient traffic and turnover. Using an antibacterial coating on surfaces such as patient beds, rails, desks, fabrics, and medical devices has been proposed as a cost-effective option for limiting the spread of infection [7,8,10]. This strategy could provide an added layer of protection against potential pathogens without additional detergents or disinfectants and reduce the labor required for cleaning. However, before such a practice can be widely recommended to the healthcare industry to replace cleaning personnel, it should be subjected to prospective studies to assess its efficacy from an environmental and clinical standpoint. Multi-center controlled studies should be conducted to evaluate how well the coating works to prevent the spread of infection and its impact on the environment. Furthermore, despite most of the antibacterial resistance investigations being based on in vitro studies of the triclosan particles, further research would be required to investigate the potential in developing antimicrobial resistance on triclosan coating materials.

In summary, antibacterial materials have recently gained popularity as a means of disinfection to prevent contact transmission. Among these materials, antibacterial coatings have been developed to provide a protective surface unsuitable for bacterial growth, thus preventing bacterial colonization. In our study, two different antibacterial coating formulas exhibited strong antibacterial efficacy against pathogens (R>2), and this effect persisted even after bleach cleaning. However, prolonged exposure to UV light may compromise the efficacy of the coatings. Overall, these findings suggest that antibacterial coatings have potential as tools for preventing the spread of pathogens.

## Figures and Tables

**Table 1:** (**a**) The bacterial reduction rates, R(JIS), of the antibacterial coating materials 23EV and 28SC; (**b**) The bacterial reduction rates, R(Total), of the antibacterial coating materials 23EV and 28SC.

(**a**)										
**R(JIS)**	***E. coli***	***Salmonella***	***P. aeruginosa***	***S. aureus***	***A. baumannii***	***MDRAB***	***MRSA***	***VREF***	***K. pneumoniae***	***ESBL***
23EV	5.48	4.26	3.49	2.57	4.97	3.25	3.31	4	5.43	5.07
28SC	3.48	5.23	5.67	3.14	5.17	3.01	2.14	2.12	5.3	5.32
(**b**)										
**R(Total)**	***E. coli***	***Salmonella***	***P. aeruginosa***	***S. aureus***	***A. baumannii***	***MDRAB***	***MRSA***	***VREF***	***K. pneumoniae***	***ESBL***
23EV	3.14	3.62	3.49	2.62	4.90	2.95	3.2	2.00	5.13	4.77
28SC	3.48	4.93	3.45	3.07	4.87	2.82	2.12	2.12	5.00	3.09

**Table 2:** The effect of UV light on bacterial reduction with the 23EV and 28SC coating materials after 500 h of exposure.

23EV	MDRAB	MRSA	Salmonella
R(JIS)	0.072	3.795	3.993
R(Total)	0.071	3.72	3.33
**28SC**			
R(JIS)	1.135	−0.836	4.879
R(Total)	1.13	−0.837	4.58

**Table 3:** The effect of 0.06% sodium hypochlorite on the bacterial reduction with the 23EV and 28SC coating materials.

23EV	MDRAB	MRSA	Salmonella
R(JIS)	4.44	2.76	3.34
R(Total)	3.83	2.07	2.39
**28SC**			
R(JIS)	5.28	2.78	4.14
R(Total)	4.98	2.62	3.86

## Data Availability

The original contributions presented in this study are included in the article. Further inquiries can be directed to the corresponding author.

## References

[1] Pittet D., Allegranzi B., Storr J., Nejad S.B., Dziekan G., Leotsakos A., Donaldson L. (2008). Infection control as a major World Health Organization priority for developing countries. J. Hosp. Infect..

[2] Urban-Chmiel R., Marek A., Stępień-Pyśniak D., Wieczorek K., Dec M., Nowaczek A., Osek J. (2022). Antibiotic Resistance in Bacteria—A Review. Antibiotics.

[3] Huang S.S., Datta R., Platt R. (2006). Risk of acquiring antibiotic-resistant bacteria from prior room occupants. Arch. Intern. Med..

[4] Rutala W.A., Weber D.J. (2021). Disinfection and sterilization in health care facilities: An overview and current issues. Infect. Dis. Clin. N. Am..

[5] Ananda T., Modi A., Chakraborty I., Managuli V., Mukhopadhyay C., Mazumder N. (2022). Nosocomial infections and role of nanotechnology. Bioengineering.

[6] Bäumler W., Eckl D., Holzmann T., Schneider-Brachert W. (2022). Antimicrobial coatings for environmental surfaces in hospitals: A potential new pillar for prevention strategies in hygiene. Crit. Rev. Microbiol..

[7] Chen X., Ling X., Liu G., Xiao J. (2022). Antimicrobial coating: Tracheal tube application. Int. J. Nanomed..

[8] Wei H., Song X., Liu P., Liu X., Yan X., Yu L. (2022). Antimicrobial coating strategy to prevent orthopaedic device-related infections: Recent advances and future perspectives. Biomater. Adv..

[9] McMurry L.M., Oethinger M., Levy S.B. (1998). Triclosan targets lipid synthesis. Nature.

[10] Heath R.J., Li J., Roland G.E., Rock C.O. (2000). Inhibition of the staphylococcus aureusNADPH-dependent enoyl-acyl carrier protein reductase by triclosan and hexachlorophene. J. Biol. Chem..

[11] Dann A.B., Hontela A. (2011). Triclosan: Environmental exposure, toxicity and mechanisms of action. J. Appl. Toxicol..

[12] (2010). Antibacterial Products—Test for Antibacterial Activity and Efficacy (JIS Z 2801:2010 (E)).

[13] Santos Filho P.S., Santos M., Colafranceschi A.S., Pragana A.N.d.S., Correia M.G., Simões H.H., Rocha F.A., Soggia M.E.d.V., Santos A.P.M.S., Coutinho A.d.A. (2019). Effect of using triclosan-impregnated polyglactin suture to prevent infection of saphenectomy wounds in cabg: A prospective, double-blind, randomized clinical trial. Braz. J. Cardiovasc. Surg..

[14] Gee R., Charles A., Taylor N., Darbre P. (2008). Oestrogenic and androgenic activity of triclosan in breast cancer cells. J. Appl. Toxicol..

[15] James M.O., Li W., Summerlot D.P., Rowland-Faux L., Wood C.E. (2010). Triclosan is a potent inhibitor of estradiol and estrone sulfonation in sheep placenta. Environ. Int..

[16] Paul K.B., Hedge J.M., Bansal R., Zoeller R.T., Peter R., DeVito M.J., Crofton K.M. (2012). Developmental triclosan exposure decreases maternal, fetal, and early neonatal thyroxine: A dynamic and kinetic evaluation of a putative mode-of-action. Toxicology.

[17] Veldhoen N., Skirrow R.C., Osachoff H., Wigmore H., Clapson D.J., Gunderson M.P., Van Aggelen G., Helbing C.C. (2006). The bactericidal agent triclosan modulates thyroid hormone-associated gene expression and disrupts postembryonic anuran development. Aquat. Toxicol..

[18] Balmer M.E., Poiger T., Droz C., Romanin K., Bergqvist P.-A., Müller M.D., Buser H.-R. (2004). Occurrence of methyl triclosan, a transformation product of the bactericide triclosan, in fish from various lakes in Switzerland. Environ. Sci. Technol..

[19] Food and Drug Administration (2016). FDA Issues Final Rule on Safety and Effectiveness of Antibacterial Soaps; U.S. Food and Drug Administration. https://www.fda.gov/news-events/press-announcements/fda-issues-final-rule-safety-and-effectiveness-antibacterial-soaps.

[20] Halden R.U. (2014). On the need and speed of regulating triclosan and triclocarban in the United States. Environ. Sci. Technol..

[21] Kolpin D.W., Furlong E.T., Meyer M.T., Thurman E.M., Zaugg S.D., Barber L.B., Buxton H.T. (2002). Pharmaceuticals, hormones, and other organic wastewater contaminants in US streams, 1999−2000: A national reconnaissance. Environ. Sci. Technol..

[22] Lopez-Gigosos R.M., Mariscal A., Gutierrez-Bedmar M., Real M., Mariscal-López E. (2019). Carbapenem resistance in *Acinetobacter baumannii* is associated with enhanced survival on hospital fabrics. Acta Microbiol. Immunol. Hung..

